# One Letter

**Published:** 1880-10

**Authors:** 


### Our Correspondence.

PHILADELPHIA, Sept. 11th, 1880.

DR. UP DE GRAFF :

*My Dear Sir :*—I have been intending to write you several times while on my travels this summer, but that old "thief," procrastination got hold of me and prevented. I arrived here with my family the first of the month after a pleasant summer's trip. A few days after leaving Elmira, I took my family to Brandywine Springs, intending that they should spend the summer there, but after enduring about ten days of intense heat, and having on my hands a sick wife and three sick children, with the fourth only moderately well, I concluded to give it up and start for somewhere, I had no idea where, to find a pleasant place and cool weather. We went to New York and from there to Stonington, by way of the sound. We struck a beautiful moonlight night, as soon as we got into the breezes of the Sound. My second son who was so weak that he could not walk alone, brightened up very much and in the morning seemed more like himself again. Taking the morning train for Providence, we stopped off at Niantic, engaged teams to drive us over to Quonocontaug Beach, about six miles; finding a comfortable hotel there and to all appearances a pleasant place, concluded to try it awhile; so sent over for baggage and was soon installed in this primitive watering place on the Rhode Island coast. The shores are sand stretches and rocky points. An inlet came in close to the house; back was a bay about two miles long and one wide. My family finding the place very satisfactory to them, I left in a few days for a sketching trip to the east. Leaving Boston on the steamer with a friend who accompanied me most of the time, we steamed down the bay in a dense fog. The first thing that happened to relieve the monotony of *nothing to see*, was a schooner trying to come on board of us. She managed to carry away part of the wheel house, the coat room, and run her jib-boom into the upper cabin, much to the alarm of some of the passengers. No one was hurt and no very serious damage done. After proceeding about five miles, heard the whistle of a steamer approaching. Our pilot slowed down and stopped and kept signaling. We were standing in the bow; in a few minutes the steamer loomed up out of the fog, bearing right down on us. Seeing a collision was inevitable, took my friend's arm and hurried to a safer place; she struck us on the bow, just where we had been standing, but fortunately a glancing shot, so we were not cut below water line; stove in and carried away the bulwarks for about 50 feet, broke something about our machinery. Distress signal was given and she lay near us for about an hour before we could repair sufficient to go on. It was a splendid subject for a painting—our crushed bow and the other steamer just visible in the fog, laying to. After spending a dismal day at Nahant, where we arrived safely, we took steamer next night for Mt. Desert Island. I heard so much of the grand scenery along the coast of Maine, that you may know how disappointed I was when we were unable to see more than a hundred yards all the way to Bar Harlem on account of the fog. After we arrived there it cleared away and for a week the weather was fine. This Island is certainly the most picturesque of any place I ever saw. You have mountain and sea combined. Fine trout fishing, within a mile of salt water fishing. I did not try it, for I was all for work; a friend tried it the day after we arrived, and got five trout in a pond almost a mile back ; they were fine fish, weighing from one half to one and one-half pounds each. We had a clear day coming away, and the scenery all the way to Rockland, was certainly all that has ever been spoken of it. The mountain forms, especially about Camden, are remarkably fine. The number of islands of all kinds and sizes makes the different views very charming—one will be low and flat, another with cliffs 200 ft. high, some higher, with the sea breaking against them. After leaving Rockland I did not enjoy it so much, as the wind was increasing, also the sea, making it very difficult to keep your footing, especially to one who has not on his sea legs. I made quite a number of sketches along the New England coast.

When I joined my family again on the Rhode Island shore, I found they had all been getting fat and hearty. I went in then for a good rest; started a swimming class in the islet, and brought home my wife and three of the children, right good swimmers. Blue fishing was the principal sport I indulged in, consequently I had the four fingers of both hands done up in bandages. How often I thought of you and wished for you with your rod and line. I was unprepared for that kind of sport, but will be again, should I go. A friend of Dr. Palmer's of Providence, came down for a day's fish above where we were. He was down on Saturday, and when the tide suited he went out on the rocks, captured three striped bass; the three weighed ninety-three pounds; the largest one weighed forty-seven pounds. Just think of it; that fellow took out four hundred and fifty feet of line before he could check him. He said there was but few more yards left on his reel, and he thought he was to lose all, when he turned him. Don't it make your blood tingle to think of it ? It does mine, and if I get the chance next year I shall try them for a week.

